# Probiotic Therapy as a Novel Approach for Allergic Disease

**DOI:** 10.3389/fphar.2012.00171

**Published:** 2012-09-21

**Authors:** Zheng Quan Toh, Anzela Anzela, Mimi L. K. Tang, Paul V. Licciardi

**Affiliations:** ^1^Allergy and Immune Disorders, Murdoch Childrens Research InstituteMelbourne, VIC, Australia; ^2^Royal Children’s HospitalMelbourne, VIC, Australia; ^3^Department of Paediatrics, University of MelbourneMelbourne, VIC, Australia

**Keywords:** allergy, asthma, clinical, eczema, immunomodulation, probiotic

## Abstract

The prevalence of allergic disease has increased dramatically in Western countries over the past few decades. The hygiene hypothesis, whereby reduced exposure to microbial stimuli in early life programs the immune system toward a Th2-type allergic response, is suggested to be a major mechanism to explain this phenomenon in developed populations. Such microbial exposures are recognized to be critical regulators of intestinal microbiota development. Furthermore, intestinal microbiota has an important role in signaling to the developing mucosal immune system. Intestinal dysbiosis has been shown to precede the onset of clinical allergy, possibly through altered immune regulation. Existing treatments for allergic diseases such as eczema, asthma, and food allergy are limited and so the focus has been to identify alternative treatment or preventive strategies. Over the past 10 years, a number of clinical studies have investigated the potential of probiotic bacteria to ameliorate the pathological features of allergic disease. This novel approach has stemmed from numerous data reporting the pleiotropic effects of probiotics that include immunomodulation, restoration of intestinal dysbiosis as well as maintaining epithelial barrier integrity. In this mini-review, the emerging role of probiotics in the prevention and/or treatment of allergic disease are discussed with a focus on the evidence from animal and human studies.

## Introduction

Allergic diseases have become a major public health problem over the past few decades. The prevalence of eczema (atopic dermatitis), food allergy, and asthma have all increased dramatically during this time, particularly in Western society. It is believed that between 20 and 30% of individuals living in Western countries suffer at least one form of allergic disease (Zuercher et al., 2006). The impact on health care systems and society in general is significant, with allergic disease one of the most common causes of chronic illness, hospital admissions as well as school absenteeism (Su et al., 1997). Furthermore, the emotional and psychological burden on parents and families is substantial (Beattie and Lewis-Jones, 2006). Parents of children with food allergy have reported a diminished quality of life compared to parents of children with rheumatological conditions, indicating a substantial psychological burden associated with these types of diseases (Primeau et al., 2000).

Allergic diseases are characterized by an inappropriate T-helper (Th)-2 cell immune response to environmental or food antigens (Zuercher et al., 2006). Activation of this response leads to the secretion of IL-4, IL-5, and IL-13 and the production of allergen-specific IgE. In the classical paradigm, the induction of Th2 cytokine responses also act to suppress Th1 activity (mainly through IFN-γ) which helps maintain the allergic phenotype. Stability of this Th1/Th2 balance is also regulated at the gene level through the relative functions of the GATA-3 (Th2) and T-bet (Th1) transcription factors. However, novel insights into dendritic cell (DC) and regulatory T cell (Treg) biology have revealed important critical effector functions of these populations in the control of allergic responses. Several studies have shown that allergic individuals have reduced Treg numbers and function (Shreffler et al., 2009; Palomares et al., 2010), while mutations in the Treg transcription factor FoxP3 results in severe immune-mediated diseases (Goodman et al., 2012).

The mechanisms that drive the development of allergic disease in early life are yet to be fully understood. One of the more widely recognized ideas relates to intestinal microbiota, where the composition and profile of commensal bacteria interact with the developing immune system. Such interactions can influence immune maturation, potentially leading to Th2-polarized allergic responses. As such, prophylactic or therapeutic strategies that target intestinal microbiota have been the subject of intense scientific research. This review article will discuss the critical function of intestinal microbiota and the evidence for the beneficial effects of probiotics in the prevention and/or treatment of allergic disease.

## Hygiene Hypothesis, Intestinal Microbiota, and Immune Development

Reduced exposure to microbes early in life is suggested to be one of the main mechanisms to account for the increasing prevalence of allergic diseases over the past few decades. Commonly referred to as the “hygiene hypothesis,” this was originally described by Strachan (1989) and associated reduced microbial contact with increased incidence of hay fever. Today, reduced microbial exposures (and the rise in allergic conditions) have been attributed to Western lifestyle factors such as diet, antibiotic use, vaccinations, reduced household size, and improved hygiene. Epidemiological studies have shown that children raised on farms during early life have a reduced risk of developing allergic disease such as eczema or asthma (Riedler et al., 2001), while prenatal farm exposure modulates atopic sensitization later in life (Ege et al., 2006).

The human intestinal microbiota represents the most significant microbial exposure for the developing infant. As many as 10^15^ microbes consisting of 1,000 different strains are said to colonize gastrointestinal tract (GIT; Molloy et al., 2012). Under normal conditions, these bacteria have beneficial roles to the host such as digestion, immune development, and the control of intestinal epithelial cell growth and differentiation (Martin et al., 2010). Commensal bacteria are also important in the fermentation of undigestible dietary fibers, a process which produces large quantities of short-chain fatty acids in addition to the release of essential vitamins (Cummings and Macfarlane, 1997).

Colonization by commensal bacteria occurs immediately after birth and continues throughout the first year of life (Arboleya et al., 2012). Acquisition of intestinal microbiota can be influenced initially by mode of delivery, maternal microbiota as well as host genetic factors and later by breastfeeding and other environmental factors (Penders et al., 2006; Bisgaard et al., 2009; Fallani et al., 2010; Bezirtzoglou et al., 2011; van Nimwegen et al., 2011; Azad and Kozyrskyj, 2012).

One of the most important functions that intestinal microbiota have is development of the host immune system. It provides the largest source of antigenic stimuli that assists the programming of postnatal immunity through maturation of the gut-associated lymphoid tissue (GALT) while promoting tolerogenic responses to innocuous antigens, including foods. This is primarily achieved via mucosal antigen sampling by pattern recognition receptors (PRRs) expressed on intestinal epithelial cells and innate immune cells (Rautava and Walker, 2007). These specialized receptors bind to microbial-associated molecular patterns (MAMPs) that are expressed on a variety of commensal micro-organisms (Amdekar et al., 2010). An important member of the PRRs is the Toll-like receptors (TLR) that recognize a range of MAMPS such as lipoteichoic acid (TLR2) and lipopolysaccharide (TLR4) on Gram-negative and Gram-positive bacteria, respectively (Bauer et al., 2007). These signals delivered by commensal bacteria to TLRs determine the nature of the immune response and results in a combination of regulatory and inductive effector functions involving DCs, Treg, chemokines, and cytokines to prevent Th2-type allergic responses as well as other inflammatory diseases (Braga et al., 2011; Rutella and Locatelli, 2011).

Germ-free (GF) mice have provided the best evidence for the role of microbiota on the GALT, as these mice have extensive defects in GALT development. A number of studies have shown that GF mice have reduced intraepithelial lymphocytes, Peyer’s patches with impaired germinal center development as well as fewer IgA secreting plasma cells and CD4+ T cells in the lamina propria (Bandeira et al., 1990; Macpherson et al., 2001; Martin et al., 2010). Reconstitution with various microbial species can restore GALT function in these mice, further supporting the role of microbiota in immune development (Rakoff-Nahoum et al., 2004). Furthermore, while GF mice do not develop tolerance to parenterally administered antigen owing to a lack of Treg cells, the oral introduction of microbes was able to establish tolerance (Bruzzese et al., 2006). A recent study found that GF mice had decreased pro-IL-1β levels compared to wild type, suggesting an impaired ability to promote Th17 cells essential for host defense (Shaw et al., 2012). Furthermore, reconstitution with segmented filamentous bacteria, clostridia and *Alcaligenes* was able to stimulate IgA production in GF mice (Talham et al., 1999; Umesaki and Setoyama, 2000; Obata et al., 2010). Moreover, when GF mice were given a high oral dose of ovalbumin (OVA) followed by a systemic challenge to induce tolerance, the Th2 response was not affected; however reconstitution with *Bifidobacterium infantis* in these mice was sufficient for oral tolerance to occur (Sudo et al., 1997). Also, antibiotic-treated mice had altered microbiota that was associated with an increased severity in airway inflammation as well as reduced Treg numbers in the colon (Nagler-Anderson, 2000; Russell et al., 2012). It is reported that the composition of intestinal microbiota may not be able to be restored to its pre-treatment state following antibiotic use (Blaser, 2011; Dethlefsen and Relman, 2011). These data highlight the important role of microbiota in immune development and the potential for immune dysregulation such as allergy and autoimmunity when there is intestinal dysbiosis (Sjogren et al., 2009; Larsen et al., 2010; Vijay-Kumar et al., 2010; Bisgaard et al., 2011).

A relationship between intestinal microbiota and allergic disease is well-established (Bisgaard et al., 2011; Johansson et al., 2011; van Nimwegen et al., 2011). Infants with allergic parents are at least twice more likely to develop allergic diseases than non-allergic parents (Dold et al., 1992; Bisgaard et al., 2011). Several epidemiological studies have reported that microbiota differences exist between allergic and non-allergic infants as well as between countries with high or low allergy prevalence rates (Bjorksten et al., 1999, 2001; Kalliomaki et al., 2001a; Watanabe et al., 2003). In a recent study by Johansson et al. (2011) infants from non-allergic parents were more frequently colonized by healthy lactobacilli, suggesting a role for maternal microbiota in protection from allergic disease. Other early studies have shown that healthy infants are usually colonized with infant-type *B. longum* and *B. breve* species while infants with eczema are more frequently colonized with adult-type *B. adolescentis* (He et al., 2001; Ouwehand et al., 2001). Previous studies have also found reduced microbial diversity, accompanied with lower numbers of *lactobacilli* and *bifidobacteria*, and early life colonization by *Staphyloccocus aureus* and *Clostridum difficile* were associated with the development of allergic disease later in life (Penders et al., 2007; Sjogren et al., 2009).

The ability of intestinal microbiota to influence immune development has led to novel interventions that exploit these microbiota differences in allergic individuals. In recent years, probiotic bacteria have been used with some success in preventing allergic disease in high-risk infants.

## Probiotics

The WHO/FAO (Food and Agriculture Organization of the UN) define probiotics as “live micro-organisms, which when administered in adequate amounts, confer a health benefit on the host” (WHO, 2001; Reid, 2005). The identification and use of probiotics date back to the early twentieth century (Shortt, 1999; Anukam and Reid, 2007). Professor Elie Metchnikoff, considered the grandfather of modern probiotics, observed that the regular consumption of lactic acid bacteria in fermented dairy products, such as yogurt, was associated with enhanced health and longevity in Bulgarian peasants (Anukam and Reid, 2007). At the time, it was believed that fermented milk contained lactic acid bacteria that decreased the pH of the gut and suppressed the growth of proteolytic bacteria (harmful bacteria). Since then, probiotic research as increased exponentially and the benefits of probiotics have been demonstrated in a number of studies for allergy, diarrheal diseases, and inflammatory conditions.

The history of probiotic use together with the wide availability of probiotic supplements over the counter suggests a high degree of safety in humans. Indeed, probiotics are demonstrated to be safe when given to both infants and adults (Tang, 2009). In general, probiotic bacteria need to fulfill several ideal criteria in order to elicit their beneficial effects, summarized in Table 1 (Tuomola et al., 2001). In taxonomy terms, the most commonly used probiotic bacteria are species of the genera *Lactobacillus* and *Bifidobacterium*. However, probiotic effects are strain and species-specific, and their biological activity can vary depending on the selected probiotic (Licciardi and Tang, 2011). Therefore, careful consideration should be given to the probiotic strain selected for use, and should be based on supporting *in vitro* and *in vivo* data.

**Table 1:** **Characteristics of an ideal probiotic**.

Characteristic	Functional advantage
Local GIT environment	Resistance to pH, bile, and digestive enzymes
Epithelial cell adherence	Prevent binding of pathogens or food antigens
Human origin	Increased likelihood of biological effectiveness
Anti-microbial activity	Direct toxicity to harmful bacteria, viruses, fungi, and parasites
Safety	Well-tolerated, important for clinical use

*GIT, gastrointestinal tract*.

## Probiotic Mechanism of Action

**Figure 1:**
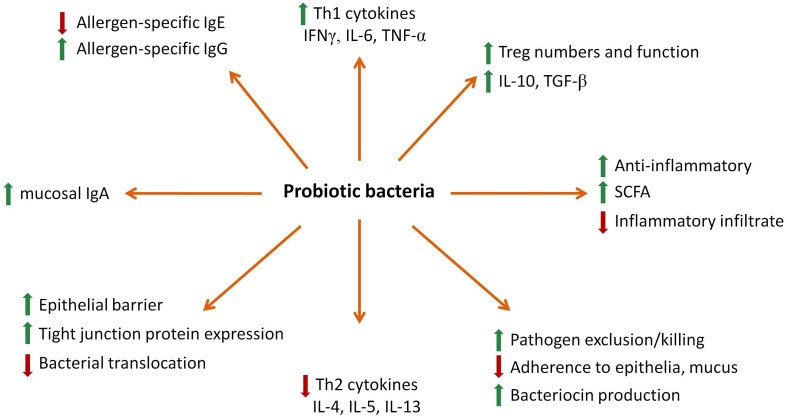
**A summary of probiotic biological effects**.

There are several mechanisms by which probiotics are proposed to exhibit beneficial effects on the host and these can be broadly classified as microbiological, epithelial, or immunological in nature (Figure 1; Oelschlaeger, 2010). Firstly, probiotic bacteria are able to modulate the composition of intestinal microbiota. It has been shown in recent studies that supplementation with probiotic bacteria such as *Lactobacillus rhamnosus* GG (LGG) or *L. casei* can modulate the composition of intestinal microbiota of allergic infants by reducing pathogenic bacteria such as *clostridia* while enhancing or maintaining beneficial *bifidobacteria* levels in the stool (Lahtinen et al., 2009; Klewicka et al., 2011). This is primarily achieved through changes in the intestinal lumen environment such as lowering the pH level and competition for nutrients that result in physiologically restrictive conditions for the growth of pathogenic bacteria (Asahara et al., 2004; Todorov et al., 2011). Probiotics can also compete with other micro-organisms for binding to specific receptors on host epithelial cells, thereby preventing potential pathogen invasion (Mukai et al., 2002; Setia et al., 2009). Transient colonization of the GIT by *Bifidobacteria* have been previously demonstrated as early as 1 week after supplementation (Langhendries et al., 1995) indicating that these effects can be induced rapidly. Moreover, *Bifidobacteria* and *Lactobacilli* probiotic treatment can modulate infant microbiota composition during early life (Mohan et al., 2006; Stratiki et al., 2007; Lahtinen et al., 2009) as well as stimulate the growth of other beneficial indigenous bacterial species in animals and humans (Tannock et al., 2000; Sui et al., 2002; Ohashi et al., 2007). Modulation of colonization by probiotic bacteria can prevent harmful pathogens from persisting in the intestinal tract, thereby facilitating clearance by the immune system. In addition, some probiotic bacteria produce bacteriocins that inhibit the growth of pathogenic bacteria (Heng et al., 2011). For example, a bacteriocin produced by *L. acidophilus* La-14 repressed the growth of *L. monocytogenes* (Todorov et al., 2011), consistent with previous findings with other probiotic species against mycobacterium (Todorov et al., 2008).

Another mechanism of probiotic action is directed at the epithelial surface where they modulate the integrity of the epithelial cell barrier and regulate the function and expression of tight junction proteins and mucus secretion (Caballero-Franco et al., 2007). The probiotic *Escherichia coli* strain Nissle 1917 was shown to increase both ZO-2 expression and PKC signaling associated with enhanced barrier function in T84 epithelial cells (Zyrek et al., 2007). Increased transepithelial resistance as well as enhanced tight junction protein phosphorylation of actinin and occludin was detected following treatment of enteroinvasive *E. coli* infected cells with live *Streptococcus thermophilus* and *Lactobacillus acidophilus* (Resta-Lenert and Barrett, 2003). Probiotics also produce significant quantities of short-chain fatty acids (SCFA) following fermentation of dietary fiber and exert potent anti-inflammatory and epithelial activities (O’Keefe et al., 2011; Macia et al., 2012). Butyrate, a common SCFA, was found to modulate the expression of certain tight junction proteins such as cingulin, ZO proteins, and occludin to improve the epithelial barrier integrity (Bordin et al., 2004; Peng et al., 2009). Another SCFA, acetate, has been shown to reduce inflammatory lesions in animal models of asthma and colitis (Maslowski et al., 2009). Neutrophils express the G protein-coupled receptors GPR41 and GPR43 that can bind SCFAs and mediate their anti-inflammatory effects (Maslowski et al., 2009). This effect for SCFAs was demonstrated through modulation of NFκB and cytokine activities *in vitro* (Tedelind et al., 2007). It has also been proposed that SCFAs exhibit histone deacetylase inhibitory properties that modify chromatin structure/function and downstream gene expression (Licciardi et al., 2010).

Various studies have found that probiotic bacteria can modulate both innate and adaptive immunity. The activation of TLRs by microbes initiates the immune response which can result in systemic and mucosal effects (Castillo et al., 2011). *Lactobacilli* attenuated pro-inflammatory responses by regulating NFκB activity (Yang et al., 2012), while other probiotics reduced TNF-α induced NFκB activation in a TLR9-dependent manner (Ghadimi et al., 2010). Probiotic bacteria also modulated DC maturation toward an anti-inflammatory IL-10 profile (Borchers et al., 2009). Moreover, human monocyte-derived DCs (MoDCs) treated with a probiotic culture supernatant released IL-10 that enabled the differentiation and survival of Treg (Rimoldi et al., 2005). *B. animalis* and *B. longum* were shown to induce IFN-γ and TNF-α release by DCs while in contrast, only *B. bifidum* could induce Th17 cell activation through the release of IL-17 by DCs (Lopez et al., 2010). LGG was also found to be a potent inducer of DC maturation while *L. delbrueckii* stimulated the secretion of both pro-inflammatory cytokines and IL-10 (Elmadfa et al., 2010).

There is a significant body of evidence demonstrating that probiotics modulate the Th1/Th2 balance to prevent the development of inflammatory diseases such as allergy. Human peripheral blood mononuclear cells (PBMCs) from allergic patients *in vitro* treated with several lactic acid bacteria including *L. plantarum*, *L. lactis*, *L. casei*, and LGG prior to stimulation with house dust mite had reduced Th2 responses characterized by lower IL-4 and IL-5 secretion (Pochard et al., 2002). Several other studies have shown similar cytokine effects, with LGG and *L. bulgaricus* inducing IL-1β, IL-6, IL-8, and TNF-α by PBMCs *in vitro* (Niers et al., 2005) while other lactic acid bacteria increased IFN-γ, TNF-α as well as IL-10 (Miettinen et al., 1998). The role of probiotics on Treg activity has also been reported. Both LGG and *B. lactis* Bb12 suppressed allergic symptoms in a mouse model of asthma by inducing TGF-β secreting Tregs (Feleszko et al., 2007). In another study, *L. acidophilus* W55 but not *L. plantarum* W62 was able to induce functional FoxP3+ Treg from CD25− cells in PBMCs from healthy adults, further supporting the species-specific effects of probiotics (de Roock et al., 2010). Other effects of probiotics that make them suitable for modulation of allergic disease include stimulation of mucosal IgA levels as well as allergen-specific B and T cell responses (Prescott and Bjorksten, 2007; Marschan et al., 2008; Maldonado Galdeano et al., 2011).

## Evidence for Probiotic Effects in Animal Models of Allergic Disease

The evaluation of probiotics in RCTs for the prevention and/or treatment of allergic disease are often the result of mechanistic data provided by *in vitro* experiments as well as animal models. Many studies have examined the use of probiotics in animal models of allergic disease, often with convincing results. Prebiotics, defined as a selectively fermented ingredient that allows specific changes in the composition and/or activity of intestinal microbiota to confer a health benefit on the host (Charalampopoulos and Rastall, 2012), are often given in combination with probiotics (termed symbiotic) however the biological effects of prebiotics are more limited and are not described in this review.

Probiotics have been shown to be effective in animal models of atopic dermatitis (eczema). Oral *L. rhamnosus* CGMCC supplementation to pregnant mice was shown to prevent the development of atopic dermatitis (eczema) when the newborns were also treated for the first 12 weeks, with reduced clinical symptoms, total plasma IgE levels and enhanced IFN-γ in skin biopsies (Curran, 2011). However, this effect was not observed when treatment was started 1 week after the onset of disease. Similar effects were observed by these authors when *L. johnsonii* NCC533 (La1) was given to mice for 4 weeks during the weaning period (Charalampopoulos and Rastall, 2012). In a dog model of atopic dermatitis, reduction in allergen-specific IgE levels were detected following treatment with LGG but no significant changes in clinical signs were observed (Cook et al., 2012). Using NC/Nga mice, several studies with various lactobacilli strains have shown beneficial effects in the prevention of atopic dermatitis-like symptoms, including increased sIgA production (Abrahamsson et al., 2012), reduced IgE (Wakabayashi et al., 2008), upregulated IL-10 (Abrahamsson et al., 2012), and reduced Th2 cytokine secretion *in vitro* (Klewicka et al., 2011), suggesting that probiotics mediate their activity through several mechanisms. In a mouse model of atopic dermatitis induced by house dust mite and dinitrochlorobenzene, treatment with a probiotic mixture containing *L. acidophilus*, *L. casei*, *L*, *reuteri*, *B. bifidum*, and *Streptococcus thermophilus* inhibited clinical progression as well as attenuating total and allergen-specific IgE levels, IL-4, IL-5, IL-10, and IL-13 levels associated with increased CD4+FoxP3+ Tregs in the ear (Klewicka et al., 2011).

The mouse allergic airway disease (AAD) model of human asthma is frequently used for the examination of probiotic effectiveness. Hougee et al. (2010) revealed that of 6 probiotic strains tested in an OVA AAD model, *B. breve* M-16V had the most potential due to the ability to improve lung function as well as reduce eosinophil numbers, OVA-specific IgE, IL-4, IL-5, and IL-10 levels in the bronchoalveolar lavage fluid (BALF). Treatment of mice with *L. casei plantarum* Lcr35 prior to OVA sensitization was found to prevent the development of airway hyperresponsiveness (AHR), BALF eosinophils as well as total serum IgE levels compared to mice treated with Lcr35 after OVA sensitization but before OVA challenge (Yu et al., 2010). Not all probiotics have been shown to be effective however. In one study, *L. reuteri* but not *L. salivarius* was able to significantly attenuate AAD by way of reduced airway eosinophils, AHR and TNF-α, IL-5 and IL-13 levels in the BALF (Forsythe et al., 2007). Subsequent studies by these authors showed that *L. reuteri* induced CD4+CD25+FoxP3+ Treg in the spleen of treated mice that were able to prevent the development of AAD following adoptive transfer into OVA-sensitized mice (Karimi et al., 2009). These results were in support of the study by Feleszko et al. (2007) where administration of LGG but not *B. lactis* Bb12 was able to suppress allergen-induced T cell proliferation associated with upregulated TGF-β secreting T cells and FoxP3+ cells in the lymph nodes. LGG was also shown to suppress AAD in mouse offspring following maternal administration along with reduced expression of TNF-α, IL-5, and IL-10 but not IL-14 or IL-14 by splenic lymphocytes (Blumer et al., 2007). Recent evidence suggests that maternal supplementation with beneficial bacteria may induce epigenetic changes in the progeny, with evidence showing that the beneficial farm-derived *Acinetobacter lwoffii* F78 bacterium was able to prevent the development of an asthma phenotype via histone modification at the *IFNG* promoter (Brand et al., 2011).

Less data is available from animal models of food allergy although existing evidence supports a role for probiotics. In a pig model of egg allergy, *Lactococcus lactis* pre-treatment was shown to reduce clinical symptoms, lower IL-4 and IL-10 levels in mitogen-stimulated mononuclear cell supernatants but lower IgG1/IgG2 and IgE/IgG2 ratios indicating a Th1 bias compared to untreated pigs (Rupa et al., 2011). The use of the probiotic mixture VSL#3 also suppressed the allergic response following shrimp tropomyosin sensitization in mice (Schiavi et al., 2011). Reduced histamine release, symptom scores, and IgE levels were associated with VSL#3 treatment as well as lower IL-4, IL-5, IL-3 but higher IFN-γ, TGF-β, and IL-10 levels in the intestine. Other studies using the OVA model of egg allergy have demonstrated anti-allergic effects for several probiotic species including *L. acidophilus* (Kim et al., 2008; Finamore et al., 2012), *B. lactis* (Kim et al., 2008), LGG (Finamore et al., 2012), and *L. lactis* (Zuercher et al., 2012). *L. casei* Shirota was unable to suppress peanut allergic responses in rats characterized by increased levels of both IgG and IgE as well as IFN-γ and IL-4 levels secreted by *in vitro* stimulated splenic and mesenteric lymph node cells (de Jonge et al., 2008).

## Evidence for the Clinical Effects of Probiotics in Allergic Disease

Over the past 15 years, a number of studies have examined the clinical benefit of probiotics for the prevention or treatment of allergic disease. Most of these have focused on eczema since this is frequently the first manifestation of allergic disease (refer Table 2 for a summary of probiotic bacteria with beneficial effects in eczema), while a few studies have looked at other outcomes such as asthma and food allergy.

**Table 2:** **Probiotics demonstrating a beneficial effect in clinical studies of eczema**.

Type of clinical study	Probiotic
Treatment	*Lactobacillus rhamnosus* GG
	*Lactobacillus rhamnosus* HN001
	*Lactobacillus sakei* KCTC
	*Lactobacillus acidophilus* La-5
	Lactobacillus acidophilus*
	*Lactobacillus salivarius* LS01
	*Lactobacillus fermentum* VR1
	*Bifidobacterium lactis* Bb12
	*Bifidobacterium lactis* UABLA-12**
	*Bifidobacterium bifidum*
Prevention	*Lactobacillus rhamnosus* GG
	*Lactobacillus rhamnosus* LC705
	*Lactobacillus paracasei* F19
	*Bifidobacterium breve* Bb99
	Propionibacterium freudenreichii***

*In combination with B. bifidum, *L. casei*, *L. salivarius*.

**In combination with *L. acidophilus* DDS-1 and fructo-oligosaccharides (prebiotic).

***In combination with galacto-oligosaccharides (prebiotic).

### Allergy treatment studies

Table 3 summarizes the major outcomes of probiotic intervention studies for the treatment of eczema. Initial studies of probiotic treatment with LGG, *Bifidobacterium lactis* Bb12 or *B. breve* M-16V for up to 8 weeks demonstrated improved eczema symptoms in infants and children compared to placebo treatment, although these involved relatively small sample sizes (Majamaa and Isolauri, 1997; Isolauri et al., 2000; Hattori et al., 2003). In the study by Weston et al. (2005) significantly reduced eczema severity (SCORAD) was observed over an 8-week *L. fermentum* VR1-003PCC treatment period, however this was not different when compared to the placebo group. In contrast, these effects of probiotics have not been confirmed in more recent and larger scale trials (Rosenfeldt et al., 2003; Viljanen et al., 2005a; Brouwer et al., 2006; Folster-Holst et al., 2006; Sistek et al., 2006; Gruber et al., 2007). However, despite the lack of a beneficial effect in these studies, subgroup analysis revealed improved SCORAD following LGG (Viljanen et al., 2005a) or a combined *L. rhamnosus* HN001 and *B. lactis* HN019 (Sistek et al., 2006) treatment in children with atopy/IgE-associated eczema. In the study by Viljanen et al. (2005b), the clinical effects observed for LGG were associated with low IL-6 and C-reactive protein (CRP) levels indicative of low-grade inflammation; it was suggested that this may trigger anti-inflammatory responses such as elevated IgA and IL-10 to suppress the ongoing allergic/inflammatory process.

**Table 3:** **Summary of probiotic intervention studies for the treatment of eczema**.

Probiotic used	Treatment period	Major outcomes	Study
*B. breve* M-16V	4 weeks (*N* = 15 infants)	Improved allergic symptoms	Hattori et al. (2003)
		Increased *Bifidobacterium* numbers	
*B. lactis* Bb12 or LGG	8 weeks (*N* = 27 infants)	Improvement in skin condition	Isolauri et al. (2000)
LGG 5 × 10^8^ CFU/g	4 weeks (*N* = 27 infants)	Improved clinical score	Majamaa and Isolauri (1997)
*L. fermentum* VRI-033 PCC 1 × 10^9^ CFU	16 weeks (*N* = 56 infants)	Reduced SCORAD	Weston et al. (2005)
		Improved skin condition	
*L. rhamnosus* or LGG 3 × 10^8^ CFU/g	12 weeks (*N* = 50 infants)	No effect on SCORAD	Brouwer et al. (2006)
LGG 10 × 10^9^ CFU	8 weeks (*N* = 54 infants)	No difference in clinical symptoms, immunological parameters, and quality of life	Folster-Holst et al. (2006)
LGG 5 × 10^9^ CFU	12 weeks (*N* = 54 infants)	No difference in symptoms, IgE levels	Gruber et al. (2007)
*L. rhamnosus* 19070-2 and *L. reuteri* DSM122460 1 × 10^10^ CFU	6 weeks (*N* = 43 children)	Improved clinical symptoms	Rosenfeldt et al. (2003)
LGG (5 × 10^9^ CFU) or MIX (LGG, 5 × 10^9^ CFU; *L. rhamnosus* LC705, 5 × 10^9^ CFU; *B. breve* Bbi99, 2 × 10^8^ CFU; and *P. freudenreichii* ssp. shermanii JS, 2 × 10^9^ CFU)	4 weeks (*N* = 230 children)	Improved clinical symptoms in LGG group	Viljanen et al. (2005a)
*L. sakei* 5 × 10^9^ CFU	12 weeks (*N* = 75 children)	Decreased SCORAD, improved clinical effects	Woo et al. (2010)
*B. bifidum*, *L. acidophilus*, *L. casei*, and *L. salivarius* 2 × 10^9^ CFU	8 weeks (*N* = 40 children)	Reduced SCORAD, total IgE	Yesilova et al. (2012)
*B. breve* M-16V and a GOS/FOS mixture	12 weeks (*N* = 90 infants)	No difference in SCORAD	van der Aa et al. (2010)
1.3 × 10^9^ CFU/100 ml		Modulated intestinal microbiota	
*L. salivarius* and prebiotic or prebiotic alone 2 × 10^9^ CFU	8 weeks (*N* = 60 children)	Reduced SCORAD, improved clinical condition, less medication use	Wu et al. (2012)
*L. casei* 1 × 10^8^ CFU/ml	12 months (*N* = 187 children)	No difference in asthma symptoms, lower incidence of rhinitis	Giovannini et al. (2007)
*L. gasseri* A5 2 × 10^9^ CFU/capsule	8 weeks (*N* = 105 children)	Decreased clinical symptoms, reduced allergic cytokines	Chen et al. (2010)

Two studies have reported a positive effect of probiotic supplementation for the treatment of eczema. An 8-week treatment with the probiotic/prebiotic mixture containing *L. acidophilus* DDS-1, *B. lactis* UABLA-12, and fructo-oligosaccharides (FOS) was observed to reduce SCORAD to a greater extent than placebo (Gerasimov et al., 2010) while a 12-week *L. sakei* KCTC 10755BP treatment in young children also lowered SCORAD and improved mean disease activity by three-fold over placebo-treated children (Woo et al., 2010). In the recent study by Yesilova et al. (2012), children aged 1–3 years with a history of eczema were treated with a combination of *B. bifidum*, *L. acidophilus*, *L. casei*, *and L. salivarius* for 8 weeks and found reductions in SCORAD as well as serum cytokines IL-5, IL-6, IFN-γ, and total serum IgE levels, but not IL-2, IL-4, IL-10, or TNF-α compared to the placebo group. However, the use of a combined probiotic and prebiotic formulation (synbiotic) containing *B. breve* M-16V and a mixture of galacto- and fructo-oligosaccharides (GOS/FOS) were unable to improve eczema severity compared to placebo although an improved SCORAD was observed for infants with IgE-associated eczema (van der Aa et al., 2010). In a study by Wu et al. (2012), treatment of children suffering moderate to severe eczema with a combination of *L. salivarius* and FOS for 8 weeks resulted in significantly reduced severity scores compared to FOS only, although no placebo group was used for baseline comparison. A similar result was found in adults treated with *L. salivarius* LS01 for 16 weeks, with significantly lower SCORAD compared to placebo as well as decreased IFN-γ, IL-2, and Th1/Th2 cytokine ratio (Drago et al., 2011).

The evidence for a beneficial effect of probiotics in the treatment of eczema based on several systematic reviews and meta-analyses are inconclusive (Boyle et al., 2008; Lee et al., 2008; Tang et al., 2010). In particular, the Cochrane meta-analysis (Boyle et al., 2008) found no significant reduction in eczema symptoms or severity by probiotics compared to placebo. Moreover, analysis of those participants with atopy or with severe disease was not able to identify a subset of patients that may benefit from probiotic therapy. Explanations for the lack of a probiotic treatment effect could be due to the significant heterogeneity between studies, both in the study populations and the selection of probiotic strains used. It is possible that the activity of potentially beneficial probiotic bacteria may be masked in pooled analyses. Therefore, the ability of probiotics to improve eczema outcomes cannot be completely excluded.

In the context of food allergy, tolerance to food antigens during infancy is a critical step in the development of the immune system. Given the ability of probiotics to modulate mucosal responses such as IgA production, DC, and Treg numbers as well as maintaining the GIT epithelial barrier integrity, it is no surprise that they have been investigated in food allergic individuals (Isolauri et al., 1993; Majamaa and Isolauri, 1997; Pelto et al., 1998). Currently, no evidence has been shown to suggest that probiotics can induce clinical tolerance to food antigens. However, the few studies that have examined whether probiotic treatment can modify the natural course of food allergy have not demonstrated an effect. In a randomized double-blind placebo-controlled study of 119 infants with challenge confirmed cow’s milk allergy, supplementation with *L. casei* CRL431 and *B. lactis* Bb12 for 12 months did not affect acquisition of tolerance to cow’s milk (Hol et al., 2008). In another study in children less than 3 years of age sensitized to egg, peanut, or cow’s milk, treatment with a probiotic mix containing *Lactobacillus* and *Bifidobacterium* species for 3 months failed to influence sensitization (SPT size or allergen-specific IgE levels) or *ex vivo* immune responses (Flinterman et al., 2007). In addition, one study that examined SCORAD outcomes in children with eczema and cow’s milk allergy also found no effect of probiotic treatment (Viljanen et al., 2005a).

In contrast to those studies on eczema outcomes, there are limited studies on the effect of probiotic treatment for asthma. In a small study, *L. acidophilus* treatment had no impact on clinical asthma in adults (Wheeler et al., 1997). Furthermore, adults given *B. breve* M-16V and GOS/FOS (prebiotic) did not show any improvement in lung function or bronchial inflammation although IL-5 levels were reduced (van de Pol et al., 2011). A complicating factor is that several studies include mixed populations involving patients with asthma and allergic rhinitis rather than either condition alone. This makes demonstration of any real benefit more difficult. Indeed, while no benefit was observed in studies involving heterogeneous populations treated with *L. casei* (Giovannini et al., 2007) or LGG (Helin et al., 2002), one study of children with asthma/allergic rhinitis treated with *L. gasseri* showed significant improvements in clinical symptoms as well as reduced allergic cytokine levels such IL-13 (Chen et al., 2010). In summary, the conflicting data from the few reported studies do not support the use of probiotics for the treatment of asthma.

### Allergy prevention studies

Prevention of allergic disease remains the greatest challenge for clinicians. While a paucity and evidence – mainly inconclusive – exists for the use of probiotics in the treatment of allergic disease, several clinical trials have been successful in the use of probiotics for the prevention of allergic disease. At present, a total of 14 randomized controlled trials evaluating various probiotics have been reported, mostly involving infants of families with a history of allergic disease (summarized in Table 4; Kalliomaki et al., 2001b; Rautava et al., 2006; Abrahamsson et al., 2007; Kukkonen et al., 2007; Taylor et al., 2007; Huurre et al., 2008; Kopp et al., 2008; Wickens et al., 2008; Niers et al., 2009; Soh et al., 2009; West et al., 2009; Dotterud et al., 2010; Kim et al., 2010; Boyle et al., 2011).

**Table 4:** **Summary of probiotic intervention studies for the prevention of eczema**.

Probiotic used	Treatment period	Outcomes	Study
LGG (1.8 × 10^10^ CFU/day)	Prenatal: 36 weeks until delivery (*N* = 212)	No effect on eczema, IgE-associated eczema, and sensitization at 12 months of age	Boyle et al. (2011)
*L. rhamnosus* LPR (2 × 10^7^ CFU/day) and *B. longum* BL999 (1 × 10^7^ CFU/day)	Postnatal: 0–6 months (*N* = 253)	No difference in incidence of eczema, sensitization, and IgE levels at 12 months of age	Soh et al. (2009)
LGG and *B. lactis* Bb12 (1 × 10^10^ CFU/day)	Postnatal: 0–12 months (*N* = 72)	No difference in eczema, sensitization at 12 months of age	Rautava et al. (2006)
		Increased cow’s milk-specific IgA secreting cells	
*L. acidophilus* LAVRI-A1 (3 × 10^10^ CFU/day)	Postnatal: 0–6 months (*N* = 172)	No difference in eczema, increased sensitization, and wheezing	Taylor et al. (2007)
*L. paracasei* F19 (1 × 10^8^ CFU/day) or placebo	Postnatal: 6–15 months (*N* = 179)	Decreased in eczema, no difference in asthma, IgE levels, or sensitization	West et al. (2009)
LGG (1 × 10^10^ CFU/day)	Pre- and postnatal: 2–4 weeks before delivery; 0–6 months after delivery (*N* = 132)	Decreased atopic eczema, no difference in IgE, and skin prick test result at 2 years of age	Kalliomaki et al. (2001b)
MIX (LGG 5 × 10^9^ CFU/day), *L. rhamnosus* LC705 (5 × 10^9^ CFU/day), *B. breve* Bb99 (2 × 10^8^ CFU/day), *P. freudenreichii* ssp Shermani JS (2 × 10^9^ CFU/day), and prebiotic	Pre- and postnatal: 2–4 weeks before delivery; 0–6 months after delivery (*N* = 925)	Reduction in eczema and IgE-associated eczema, no difference in other allergic outcomes by 2 years of age	Kukkonen et al. (2007)
L. *reuteri* ATCC 55730 (1 × 10^10^ CFU/day)	Pre- and postnatal: 4 weeks before delivery; 0–12 months after delivery (*N* = 188)	No difference in incidence of eczema, reduced IgE-associated eczema, and sensitization in infants by 2 years of age	Abrahamsson et al. (2007)
LGG (5 × 10^10^ CFU/day)	Pre- and postnatal: 4–6 weeks before delivery; 0–6 months (0–3 months to breastfeeding mothers, 3–6 months to infants; *N* = 94)	No effect on eczema or sensitization, increased recurrent episodes of wheezing bronchitis at 2 years of age	Kopp et al. (2008)
*L. rhamnosus* HN001 (6 × 10^9^ CFU/day) or *B. animalis* subsp. *lactis* HN019 (9 × 10^9^ CFU/day)	Pre- and postnatal: 5 weeks until delivery; 0–6 months to breastfeeding mothers and 2 years to infants (*N* = 474)	Reduced eczema in both groups, reduced IgE-associated eczema (HN001 only), no effect on sensitization in either group at 2 years of age	Wickens et al. (2008)
LGG, *L. acidophilus* La-5 and *B. lactis* Bb12 (1 × 10^9^ CFU/day)	Pre- and postnatal: 36 weeks until delivery, 0–3 months to breastfeeding mothers (*N* = 278)	Reduced eczema, no effect on asthma or sensitization	Dotterud et al. (2010)
LGG or *B. lactis* Bb12 (1 × 10^10^ CFU/day)	Pre- and postnatal: first trimester until delivery; until end of exclusive breastfeeding (*N* = 140)	No effect on eczema and sensitization, less sensitization in infants of allergic mothers at 1 year of age (*B. lactis* group)	Huurre et al. (2008)
MIX (*B. bifidum* BGN4, *B. lactis* AD011, and *L. acidophilus* AD031; all 1.6 × 10^9^ CFU/day)	Pre- and postnatal: 4–8 weeks before delivery; 0–3 months to mothers and 4–6 months to infants (*N* = 112)	Reduced eczema, no difference in total IgE, or sensitization	Kim et al. (2010)
MIX (*B*. *bifidum* W23, *B. lactis* W52 and *L. lactis* W58) (all 1 × 10^9^ CFU/day)	Pre- and postnatal: 6 weeks before delivery; 0–12 months to infants (*N* = 102)	Lower parental-reported eczema for the first 3 months, no difference after 3 months	Niers et al. (2009)

Timing and duration of the probiotic intervention appears to be major factor in determining their beneficial effects. Nine of the 14 RCTs involved both a prenatal and postnatal intervention period, while four studies evaluated only postnatal and one study examined a prenatal only approach (Boyle et al., 2011). For the combined prenatal/postnatal probiotic studies, there was a significant reduction in the cumulative incidence of eczema and/or IgE-associated eczema in six of the nine published RCTs (Kalliomaki et al., 2001b; Kukkonen et al., 2007; Wickens et al., 2008; Niers et al., 2009; Dotterud et al., 2010; Kim et al., 2010) by age 2 years. No such effects were reported in the other three studies (Abrahamsson et al., 2007; Huurre et al., 2008; Kopp et al., 2008). Interestingly, of these nine prenatal/postnatal studies, a beneficial effect was found in three of five studies that used LGG with or without other probiotics (LGG (Kalliomaki et al., 2001b); *L. rhamnosus* LC705, *B. breve* Bb99, and *Propionibacterium freudenreichii*+ the prebiotic GOS (Kukkonen et al., 2007); LGG, *L. acidophilus* La-5 and *B. lactis* Bb12 (Dotterud et al., 2010). In the other two studies using LGG, one was a study in infants irrespective of a family history of allergic disease (Huurre et al., 2008) while the other study by Kopp et al. (2008) found no effect despite utilizing a similar protocol and LGG dose to that of the landmark study by Kalliomaki et al. (2001b). A recent meta-analysis of the impact of maternal probiotic supplementation on eczema development found a significantly reduced risk in children by 2–7 years of age with the use of lactobacilli but not with other probiotic species and strains compared to placebo treatment (Doege et al., 2012). The remaining four prenatal/postnatal RCTs used a variety of probiotic species or combinations with varying efficacy. Only the study by Abrahamsson et al. (2007) showed no difference in eczema, sensitization, or other allergic diseases at 2 years following *L. reuteri* treatment. Two studies using different probiotic mixtures reported a reduction in eczema at 1 year (Kim et al., 2010) and 3 months (Niers et al., 2009) – however in this latter study, the effect at 3 months did not persist and the outcomes were parental-reported, not clinician assessed. Wickens et al. (2008) investigated the effects of two different probiotic interventions in relation to placebo and reported that *L. rhamnosus* HN001 but not *B. animalis* subspecies *lactis* HN013 was able to significantly reduce both eczema and IgE-associated eczema at 2 years. Both of these treatments however had no impact on sensitization status. This supports the view that not all probiotics are the same such that they may elicit unique biological activities. Therefore, selection of probiotic species and strains should be carefully considered in the design of future clinical trials. It is important to note that despite the beneficial effects of probiotics in some of the studies, there were also increased risks of asthma-like symptoms at 2 years in the study by Kopp et al. (2008) and at 7 years in the study by Kalliomaki et al. (2007), suggesting that it will be critical to follow these cohorts for several years to determine any long-term impact (beneficial or otherwise) of the probiotic effect.

No beneficial effects on eczema or sensitization were found in three of the four studies using a postnatal treatment approach (Rautava et al., 2006; Taylor et al., 2007; Soh et al., 2009) with one study that used *L. acidophilus* LAVRI-A1 demonstrating an increased risk of both IgE-associated eczema and atopic sensitization at 1 year of age (Taylor et al., 2007). The remaining study found a reduced cumulative incidence of eczema at 13 months with *L. paracasei* F19 (West et al., 2009). Overall, these studies suggest that a postnatal probiotic treatment alone may be insufficient in reducing the clinical symptoms of allergic disease and highlights that the importance of the early life period in modulating microbiota and/or immune function begins prior to birth. In addition, the differences in study designs make it difficult to draw meaningful conclusions as two studies recruiting high-risk infants with a family history of allergic disease (Taylor et al., 2007; Soh et al., 2009) while the other two studies included formula-fed infants irrespective of allergic disease family history (Rautava et al., 2006; West et al., 2009). In contrast, the only study to date that evaluated a prenatal only probiotic approach (using LGG) showed no evidence for a beneficial effect on eczema or sensitization at 12 months (Boyle et al., 2011). This suggests that a prenatal alone intervention is insufficient and that inclusion of a postnatal treatment period is required. Alternatively, probiotic species/strains other than LGG may be effective for the prevention of eczema.

Breastfeeding is one parameter that may be critical in mediating probiotic effects. Infants who were breastfed by mothers treated with probiotics during pregnancy and breastfeeding benefited the most, even when probiotics were not administered directly to them (Dotterud et al., 2010). Breast milk has important immunoregulatory factors such as TGF-β and IgA which can help protect against the development of allergic disease (Rautava et al., 2006). The biological mechanisms underpinning these outcomes are not well understood and require further investigation.

In summary, the use of probiotic bacteria for the prevention and/or treatment of allergic disease have shown promising results to date. However, the validity of these findings need to be confirmed by further randomized controlled trials. In particular, for eczema, the timing of the probiotic intervention appears to be important, with a prenatal component critical for protective effects (Osborn and Sinn, 2007). Furthermore, a combined prenatal with early postnatal treatment may be equally effective (Boyle et al., 2011) while a postnatal alone approach less successful. From the existing literature, it is clear that several variables can have an impact on the potential beneficial effects observed, including the species and strain of probiotic used, geographical differences in the populations studied as well as the dose and duration of administration. These differences make drawing conclusions about the effectiveness of probiotics more difficult. Further studies are therefore required to determine the optimal dose, bacterial strain(s), timing for intervention, and patient populations that would provide optimal effects in the prevention and/or treatment of allergic disease.

## Conclusion

Studies suggest a potential role for selected probiotics in the prevention of eczema, especially IgE-associated eczema. The efficacy of probiotics for the treatment of allergic disease however requires further examination. Careful selection of appropriate probiotic bacteria for future studies will be important, which may be aided by *in vitro*, preclinical and pilot studies. In addition, there is insufficient evidence that probiotics may be of benefit for the prevention of other allergic conditions. An important limitation of meta-analyses of studies evaluating probiotics for the prevention of allergic diseases is that data have been pooled from studies conducted with a variety of different probiotic combinations. Further clinical studies using the most effective study designs incorporating prenatal/postnatal treatment and selection of appropriate probiotic bacteria are required to validate the findings of earlier studies. Understanding the mechanisms of protection in allergic disease will greatly assist in developing more targeted strategies for the prevention or treatment of allergic disease.

## Conflict of Interest Statement

The authors declare that the research was conducted in the absence of any commercial or financial relationships that could be construed as a potential conflict of interest.

## References

[B1] Abrahamsson T. R. Jakobsson H. E. Andersson A. F. Bjorksten B. Engstrand L. Jenmalm M. C. (2012). Low diversity of the gut microbiota in infants with atopic eczema. J. Allergy Clin. Immunol. 129, 434–440, 440 e1–e2. 10.1016/j.jaci.2011.10.025 22153774

[B2] Abrahamsson T. R. Jakobsson T. Bottcher M. F. Fredrikson M. Jenmalm M. C. Bjorksten B. Oldaeus G. (2007). Probiotics in prevention of IgE-associated eczema: a double-blind, randomized, placebo-controlled trial. J. Allergy Clin. Immunol. 119, 1174–1180 10.1016/j.jaci.2007.01.007 17349686

[B3] Amdekar S. Dwivedi D. Roy P. Kushwah S. Singh V. (2010). Probiotics: multifarious oral vaccine against infectious traumas. FEMS Immunol. Med. Microbiol. 58, 299–306 20100178 10.1111/j.1574-695X.2009.00630.x

[B4] Anukam K. C. Reid G. (2007). “Probiotics: 100 years (1907–2007) after Elie Metchnikoff’s Observation,” in Communicating Current Research and Educational Topics and Trends in Applied Microbiology, ed. Méndez-Vilas A. (Formatex.org) 466–474

[B5] Arboleya S. Binetti A. Salazar N. Fernandez N. Solis G. Hernandez-Barranco A. Margolles A. de Los Reyes-Gavilan C. G. Gueimonde M. (2012). Establishment and development of intestinal microbiota in preterm neonates. FEMS Microbiol. Ecol. 79, 763–772 10.1111/j.1574-6941.2011.01261.x 22126419

[B6] Asahara T. Shimizu K. Nomoto K. Hamabata T. Ozawa A. Takeda Y. (2004). Probiotic bifidobacteria protect mice from lethal infection with Shiga toxin-producing Escherichia coli O157:H7. Infect. Immun. 72, 2240–2247 10.1128/IAI.72.4.2240-2247.2004 15039348 PMC375161

[B7] Azad M. B. Kozyrskyj A. L. (2012). Perinatal programming of asthma: the role of gut microbiota. Clin. Dev. Immunol. 2012, 932072 10.1155/2012/932072 22110540 PMC3216351

[B8] Bandeira A. Mota-Santos T. Itohara S. Degermann S. Heusser C. Tonegawa S. Coutinho A. (1990). Localization of gamma/delta T cells to the intestinal epithelium is independent of normal microbial colonization. J. Exp. Med. 172, 239–244 10.1084/jem.172.1.239 2141628 PMC2188170

[B9] Bauer S. Hangel D. Yu P. (2007). Immunobiology of toll-like receptors in allergic disease. Immunobiology 212, 521–533 10.1016/j.imbio.2007.03.011 17544836

[B10] Beattie P. E. Lewis-Jones M. S. (2006). A comparative study of impairment of quality of life in children with skin disease and children with other chronic childhood diseases. Br. J. Dermatol. 155, 145–151 10.1111/j.1365-2133.2006.07525.x 16792766

[B11] Bezirtzoglou E. Tsiotsias A. Welling G. W. (2011). Microbiota profile in feces of breast- and formula-fed newborns by using fluorescence in situ hybridization (FISH). Anaerobe 17, 478–482 10.1016/j.anaerobe.2011.03.009 21497661

[B12] Bisgaard H. Halkjaer L. B. Hinge R. Giwercman C. Palmer C. Silveira L. Strand M. (2009). Risk analysis of early childhood eczema. J. Allergy Clin. Immunol. 123, 1355–1360 e5. 10.1016/j.jaci.2009.03.046 19501236

[B13] Bisgaard H. Li N. Bonnelykke K. Chawes B. L. Skov T. Paludan-Muller G. Stokholm J. Smith B. Krogfelt K. A. (2011). Reduced diversity of the intestinal microbiota during infancy is associated with increased risk of allergic disease at school age. J. Allergy Clin. Immunol. 128, 646–652 e1–e5. 10.1016/j.jaci.2011.04.060 21782228

[B14] Bjorksten B. Naaber P. Sepp E. Mikelsaar M. (1999). The intestinal microflora in allergic Estonian and Swedish 2-year-old children. Clin. Exp. Allergy 29, 342–346 10.1046/j.1365-2222.1999.00560.x 10202341

[B15] Bjorksten B. Sepp E. Julge K. Voor T. Mikelsaar M. (2001). Allergy development and the intestinal microflora during the first year of life. J. Allergy Clin. Immunol. 108, 516–520 10.1067/mai.2001.118130 11590374

[B16] Blaser M. (2011). Antibiotic overuse: stop the killing of beneficial bacteria. Nature 476, 393–394 10.1038/476393a 21866137

[B17] Blumer N. Sel S. Virna S. Patrascan C. C. Zimmermann S. Herz U. Renz H. Garn H. (2007). Perinatal maternal application of Lactobacillus rhamnosus GG suppresses allergic airway inflammation in mouse offspring. Clin. Exp. Allergy 37, 348–357 10.1111/j.1365-2222.2007.02671.x 17359385

[B18] Borchers A. T. Selmi C. Meyers F. J. Keen C. L. Gershwin M. E. (2009). Probiotics and immunity. J. Gastroenterol. 44, 26–46 10.1007/s00535-008-2296-0 19159071

[B19] Bordin M. D’Atri F. Guillemot L. Citi S. (2004). Histone deacetylase inhibitors up-regulate the expression of tight junction proteins. Mol. Cancer Res. 2, 692–701 15634758

[B20] Boyle R. J. Bath-Hextall F. J. Leonardi-Bee J. Murrell D. F. Tang M. L. (2008). Probiotics for treating eczema. Cochrane Database Syst. Rev. 4, CD006135 18843705 10.1002/14651858.CD006135.pub2

[B21] Boyle R. J. Ismail I. H. Kivivuori S. Licciardi P. V. Robins-Browne R. M. Mah L. J. Axelrad C. Moore S. Donath S. Carlin J. B. Lahtinen S. J. Tang M. L. (2011). Lactobacillus GG treatment during pregnancy for the prevention of eczema: a randomized controlled trial. Allergy 66, 509–516 10.1111/j.1398-9995.2010.02507.x 21121927

[B22] Braga M. Quecchia C. Cavallucci E. Di Giampaolo L. Schiavone C. Petrarca C. Di Gioacchino M. (2011). T regulatory cells in allergy. Int. J. Immunopathol. Pharmacol. 24(Suppl. 1), 55S–64S 21329567

[B23] Brand S. Teich R. Dicke T. Harb H. Yildirim A. O. Tost J. Schneider-Stock R. Waterland R. A. Bauer U. M. von Mutius E. Garn H. Pfefferle P. I. Renz H. (2011). Epigenetic regulation in murine offspring as a novel mechanism for transmaternal asthma protection induced by microbes. J. Allergy Clin. Immunol. 128, 618–625 e1–e7. 10.1016/j.jaci.2011.04.035 21680015

[B24] Brouwer M. L. Wolt-Plompen S. A. Dubois A. E. van der Heide S. Jansen D. F. Hoijer M. A. Kauffman H. F. Duiverman E. J. (2006). No effects of probiotics on atopic dermatitis in infancy: a randomized placebo-controlled trial. Clin. Exp. Allergy 36, 899–906 10.1111/j.1365-2222.2006.02513.x 16839405

[B25] Bruzzese E. Volpicelli M. Squaglia M. Tartaglione A. Guarino A. (2006). Impact of prebiotics on human health. Dig. Liver Dis. 38(Suppl. 2), S283–S287 10.1016/j.dld.2005.10.012 17259092

[B26] Caballero-Franco C. Keller K. De Simone C. Chadee K. (2007). The VSL#3 probiotic formula induces mucin gene expression and secretion in colonic epithelial cells. Am. J. Physiol. Gastrointest. Liver Physiol. 292, G315–G322 10.1152/ajpgi.00265.2006 16973917

[B27] Castillo N. A. Perdigon G. de Moreno de Leblanc A. (2011). Oral administration of a probiotic Lactobacillus modulates cytokine production and TLR expression improving the immune response against Salmonella enterica serovar Typhimurium infection in mice. BMC Microbiol. 11, 177 10.1186/1471-2180-11-177 21813005 PMC3173335

[B28] Charalampopoulos D. Rastall R. A. (2012). Prebiotics in foods. Curr. Opin. Biotechnol. 23, 187–191 10.1016/j.copbio.2011.12.028 22244693

[B29] Chen Y. S. Jan R. L. Lin Y. L. Chen H. H. Wang J. Y. (2010). Randomized placebo-controlled trial of lactobacillus on asthmatic children with allergic rhinitis. Pediatr. Pulmonol. 45, 1111–1120 10.1002/ppul.21264 20658483

[B30] Cook M. T. Tzortzis G. Charalampopoulos D. Khutoryanskiy V. V. (2012). Microencapsulation of probiotics for gastrointestinal delivery. J. Control. Release. 162, 56–67 10.1016/j.jconrel.2012.06.003 22698940

[B31] Cummings J. H. Macfarlane G. T. (1997). Role of intestinal bacteria in nutrient metabolism. JPEN J. Parenter. Enteral Nutr. 21, 357–365 10.1177/0148607197021006357 9406136

[B32] Curran A. D. (2011). Enabling a better working Britain: celebrating the centenary of the Health and Safety Laboratory. Occup. Med. (Chic. Ill) 61, 290–291 10.1093/occmed/kqr055 21831808

[B33] de Jonge J. D. Ezendam J. Knippels L. M. Penninks A. H. Pieters R. van Loveren H. (2008). Lactobacillus casei Shirota does not decrease the food allergic response to peanut extract in Brown Norway rats. Toxicology 249, 140–145 10.1016/j.tox.2008.04.016 18524449

[B34] de Roock S. van Elk M. van Dijk M. E. Timmerman H. M. Rijkers G. T. Prakken B. J. Hoekstra M. O. de Kleer I. M. (2010). Lactic acid bacteria differ in their ability to induce functional regulatory T cells in humans. Clin. Exp. Allergy 40, 103–110 19817754 10.1111/j.1365-2222.2009.03344.x

[B35] Dethlefsen L. Relman D. A. (2011). Incomplete recovery and individualized responses of the human distal gut microbiota to repeated antibiotic perturbation. Proc. Natl. Acad. Sci. U.S.A. 108(Suppl. 1), 4554–4561 10.1073/pnas.1000087107 20847294 PMC3063582

[B36] Doege K. Grajecki D. Zyriax B. C. Detinkina E. Zu Eulenburg C. Buhling K. J. (2012). Impact of maternal supplementation with probiotics during pregnancy on atopic eczema in childhood – a meta-analysis. Br. J. Nutr. 107, 1–6 10.1017/S0007114511007094 21787448

[B37] Dold S. Wjst M. von Mutius E. Reitmeir P. Stiepel E. (1992). Genetic risk for asthma, allergic rhinitis, and atopic dermatitis. Arch. Dis. Child. 67, 1018–1022 10.1136/adc.67.8.1018 1520004 PMC1793604

[B38] Dotterud C. K. Storro O. Johnsen R. Oien T. (2010). Probiotics in pregnant women to prevent allergic disease: a randomized, double-blind trial. Br. J. Dermatol. 163, 616–623 10.1111/j.1365-2133.2010.09889.x 20545688

[B39] Drago L. Iemoli E. Rodighiero V. Nicola L. De Vecchi E. Piconi S. (2011). Effects of Lactobacillus salivarius LS01 (DSM 22775) treatment on adult atopic dermatitis: a randomized placebo-controlled study. Int. J. Immunopathol. Pharmacol. 24, 1037–1048 22230409 10.1177/039463201102400421

[B40] Ege M. J. Bieli C. Frei R. van Strien R. T. Riedler J. Ublagger E. Schram-Bijkerk D. Brunekreef B. van Hage M. Scheynius A. Pershagen G. Benz M. R. Lauener R. von Mutius E. Braun-Fahrlander C. (2006). Prenatal farm exposure is related to the expression of receptors of the innate immunity and to atopic sensitization in school-age children. J. Allergy Clin. Immunol. 117, 817–823 10.1016/j.jaci.2005.12.1307 16630939

[B41] Elmadfa I. Klein P. Meyer A. L. (2010). Immune-stimulating effects of lactic acid bacteria in vivo and in vitro. Proc. Nutr. Soc. 69, 416–420 10.1017/S002966511000279X 20550748

[B42] Fallani M. Young D. Scott J. Norin E. Amarri S. Adam R. Aguilera M. Khanna S. Gil A. Edwards C. A. Dore J. (2010). Intestinal microbiota of 6-week-old infants across Europe: geographic influence beyond delivery mode, breast-feeding, and antibiotics. J. Pediatr. Gastroenterol. Nutr. 51, 77–84 10.1097/MPG.0b013e3181d1b11e 20479681

[B43] Feleszko W. Jaworska J. Rha R. D. Steinhausen S. Avagyan A. Jaudszus A. Ahrens B. Groneberg D. A. Wahn U. Hamelmann E. (2007). Probiotic-induced suppression of allergic sensitization and airway inflammation is associated with an increase of T regulatory-dependent mechanisms in a murine model of asthma. Clin. Exp. Allergy 37, 498–505 10.1111/j.1365-2222.2006.02629.x 17430345

[B44] Finamore A. Roselli M. Britti M. S. Merendino N. Mengheri E. (2012). Lactobacillus rhamnosus GG and Bifidobacterium animalis MB5 induce intestinal but not systemic antigen-specific hyporesponsiveness in ovalbumin-immunized rats. J. Nutr. 142, 375–381 10.3945/jn.111.148924 22223570

[B45] Flinterman A. E. Knol E. F. van Ieperen-van Dijk A. G. Timmerman H. M. Knulst A. C. Bruijnzeel-Koomen C. A. Pasmans S. G. van Hoffen E. (2007). Probiotics have a different immunomodulatory potential in vitro versus ex vivo upon oral administration in children with food allergy. Int. Arch. Allergy Immunol. 143, 237–244 10.1159/000099467 17290150

[B46] Folster-Holst R. Muller F. Schnopp N. Abeck D. Kreiselmaier I. Lenz T. von Ruden U. Schrezenmeir J. Christophers E. Weichenthal M. (2006). Prospective, randomized controlled trial on Lactobacillus rhamnosus in infants with moderate to severe atopic dermatitis. Br. J. Dermatol. 155, 1256–1261 10.1111/j.1365-2133.2006.07558.x 17107398

[B47] Forsythe P. Inman M. D. Bienenstock J. (2007). Oral treatment with live Lactobacillus reuteri inhibits the allergic airway response in mice. Am. J. Respir. Crit. Care Med. 175, 561–569 10.1164/rccm.200606-821OC 17204726

[B48] Gerasimov S. V. Vasjuta V. V. Myhovych O. O. Bondarchuk L. I. (2010). Probiotic supplement reduces atopic dermatitis in preschool children: a randomized, double-blind, placebo-controlled, clinical trial. Am. J. Clin. Dermatol. 11, 351–361 10.2165/11531420-000000000-00000 20642296

[B49] Ghadimi D. Vrese M. Heller K. J. Schrezenmeir J. (2010). Effect of natural commensal-origin DNA on toll-like receptor 9 (TLR9) signaling cascade, chemokine IL-8 expression, and barrier integritiy of polarized intestinal epithelial cells. Inflamm. Bowel Dis. 16, 410–427 19714766 10.1002/ibd.21057

[B50] Giovannini M. Agostoni C. Riva E. Salvini F. Ruscitto A. Zuccotti G. V. Radaelli G. (2007). A randomized prospective double-blind controlled trial on effects of long-term consumption of fermented milk containing Lactobacillus casei in pre-school children with allergic asthma and/or rhinitis. Pediatr. Res. 62, 215–220 10.1203/PDR.0b013e3180a76d94 17597643

[B51] Goodman W. A. Cooper K. D. McCormick T. S. (2012). Regulation generation: the suppressive functions of human regulatory T cells. Crit. Rev. Immunol. 32, 65–79 10.1615/CritRevImmunol.v32.i1.40 22428855 PMC3413266

[B52] Gruber C. Wendt M. Sulser C. Lau S. Kulig M. Wahn U. Werfel T. Niggemann B. (2007). Randomized, placebo-controlled trial of Lactobacillus rhamnosus GG as treatment of atopic dermatitis in infancy. Allergy 62, 1270–1276 10.1111/j.1398-9995.2007.01543.x 17919141

[B53] Hattori K. Yamamoto A. Sasai M. Taniuchi S. Kojima T. Kobayashi Y. Iwamoto H. Namba K. Yaeshima T. (2003). Effects of administration of bifidobacteria on fecal microflora and clinical symptoms in infants with atopic dermatitis. Arerugi 52, 20–30 12598719

[B54] He F. Ouwehand A. C. Isolauri E. Hashimoto H. Benno Y. Salminen S. (2001). Comparison of mucosal adhesion and species identification of bifidobacteria isolated from healthy and allergic infants. FEMS Immunol. Med. Microbiol. 30, 43–47 10.1111/j.1574-695X.2001.tb01548.x 11172990

[B55] Helin T. Haahtela S. Haahtela T. (2002). No effect of oral treatment with an intestinal bacterial strain, Lactobacillus rhamnosus (ATCC 53103), on birch-pollen allergy: a placebo-controlled double-blind study. Allergy 57, 243–246 10.1034/j.1398-9995.2002.1s3299.x 11906339

[B56] Heng N. C. Haji-Ishak N. S. Kalyan A. Wong A. Y. Lovric M. Bridson J. M. Artamonova J. Stanton J. A. Wescombe P. A. Burton J. P. Cullinan M. P. Tagg J. R. (2011). Genome sequence of the bacteriocin-producing oral probiotic Streptococcus salivarius strain M18. J. Bacteriol. 193, 6402–6403 10.1128/JB.06001-11 22038965 PMC3209194

[B57] Hol J. van Leer E. H. Elink Schuurman B. E. de Ruiter L. F. Samsom J. N. Hop W. Neijens H. J. de Jongste J. C. Nieuwenhuis E. E. (2008). The acquisition of tolerance toward cow’s milk through probiotic supplementation: a randomized, controlled trial. J. Allergy Clin. Immunol. 121, 1448–1454 10.1016/j.jaci.2008.03.018 18436293

[B58] Hougee S. Vriesema A. J. Wijering S. C. Knippels L. M. Folkerts G. Nijkamp F. P. Knol J. Garssen J. (2010). Oral treatment with probiotics reduces allergic symptoms in ovalbumin-sensitized mice: a bacterial strain comparative study. Int. Arch. Allergy Immunol. 151, 107–117 10.1159/000236000 19752564

[B59] Huurre A. Laitinen K. Rautava S. Korkeamaki M. Isolauri E. (2008). Impact of maternal atopy and probiotic supplementation during pregnancy on infant sensitization: a double-blind placebo-controlled study. Clin. Exp. Allergy 38, 1342–1348 10.1111/j.1365-2222.2008.03089_1.x 18477013

[B60] Isolauri E. Arvola T. Sutas Y. Moilanen E. Salminen S. (2000). Probiotics in the management of atopic eczema. Clin. Exp. Allergy 30, 1604–1610 10.1046/j.1365-2222.2000.00943.x 11069570

[B61] Isolauri E. Majamaa H. Arvola T. Rantala I. Virtanen E. Arvilommi H. (1993). Lactobacillus casei strain GG reverses increased intestinal permeability induced by cow milk in suckling rats. Gastroenterology 105, 1643–1650 8253341 10.1016/0016-5085(93)91059-q

[B62] Johansson M. A. Sjogren Y. M. Persson J. O. Nilsson C. Sverremark-Ekstrom E. (2011). Early colonization with a group of Lactobacilli decreases the risk for allergy at five years of age despite allergic heredity. PLoS ONE 6, e23031 10.1371/journal.pone.0023031 21829685 PMC3148229

[B63] Kalliomaki M. Kirjavainen P. Eerola E. Kero P. Salminen S. Isolauri E. (2001a). Distinct patterns of neonatal gut microflora in infants in whom atopy was and was not developing. J. Allergy Clin. Immunol. 107, 129–134 10.1067/mai.2001.111237 11150002

[B64] Kalliomaki M. Salminen S. Arvilommi H. Kero P. Koskinen P. Isolauri E. (2001b). Probiotics in primary prevention of atopic disease: a randomised placebo-controlled trial. Lancet 357, 1076–1079 10.1016/S0140-6736(00)04259-8 11297958

[B65] Kalliomaki M. Salminen S. Poussa T. Isolauri E. (2007). Probiotics during the first 7 years of life: a cumulative risk reduction of eczema in a randomized, placebo-controlled trial. J. Allergy Clin. Immunol. 119, 1019–1021 10.1016/j.jaci.2006.12.608 17289135

[B66] Karimi K. Inman M. D. Bienenstock J. Forsythe P. (2009). Lactobacillus reuteri-induced regulatory T cells protect against an allergic airway response in mice. Am. J. Respir. Crit. Care Med. 179, 186–193 10.1164/rccm.200806-951OC 19029003

[B67] Kim J. Y. Choi Y. O. Ji G. E. (2008). Effect of oral probiotics (Bifidobacterium lactis AD011 and Lactobacillus acidophilus AD031) administration on ovalbumin-induced food allergy mouse model. J. Microbiol. Biotechnol. 18, 1393–1400 18756099

[B68] Kim J. Y. Kwon J. H. Ahn S. H. Lee S. I. Han Y. S. Choi Y. O. Lee S. Y. Ahn K. M. Ji G. E. (2010). Effect of probiotic mix (Bifidobacterium bifidum, Bifidobacterium lactis, Lactobacillus acidophilus) in the primary prevention of eczema: a double-blind, randomized, placebo-controlled trial. Pediatr. Allergy Immunol. 21(2 Pt 2), e386–e393 10.1111/j.1399-3038.2009.00958.x 19840300

[B69] Klewicka E. Cukrowska B. Libudzisz Z. Slizewska K. Motyl I. (2011). Changes in gut microbiota in children with atopic dermatitis administered the bacteria Lactobacillus casei DN–114001. Pol. J. Microbiol. 60, 329–333 22390068

[B70] Kopp M. V. Hennemuth I. Heinzmann A. Urbanek R. (2008). Randomized, double-blind, placebo-controlled trial of probiotics for primary prevention: no clinical effects of Lactobacillus GG supplementation. Pediatrics 121, e850–e856 10.1542/peds.2007-1492 18332075

[B71] Kukkonen K. Savilahti E. Haahtela T. Juntunen-Backman K. Korpela R. Poussa T. Tuure T. Kuitunen M. (2007). Probiotics and prebiotic galacto-oligosaccharides in the prevention of allergic diseases: a randomized, double-blind, placebo-controlled trial. J. Allergy Clin. Immunol. 119, 192–198 10.1016/j.jaci.2006.12.120 17208601

[B72] Lahtinen S. J. Boyle R. J. Kivivuori S. Oppedisano F. Smith K. R. Robins-Browne R. Salminen S. J. Tang M. L. (2009). Prenatal probiotic administration can influence Bifidobacterium microbiota development in infants at high risk of allergy. J. Allergy Clin. Immunol. 123, 499–501 10.1016/j.jaci.2008.11.034 19135234

[B73] Langhendries J. P. Detry J. Van Hees J. Lamboray J. M. Darimont J. Mozin M. J. Secretin M. C. Senterre J. (1995). Effect of a fermented infant formula containing viable bifidobacteria on the fecal flora composition and pH of healthy full-term infants. J. Pediatr. Gastroenterol. Nutr. 21, 177–181 10.1097/00005176-199508000-00009 7472904

[B74] Larsen N. Vogensen F. K. van den Berg F. W. Nielsen D. S. Andreasen A. S. Pedersen B. K. Al-Soud W. A. Sorensen S. J. Hansen L. H. Jakobsen M. (2010). Gut microbiota in human adults with type 2 diabetes differs from non-diabetic adults. PLoS ONE 5, e9085 10.1371/journal.pone.0009085 20140211 PMC2816710

[B75] Lee J. Seto D. Bielory L. (2008). Meta-analysis of clinical trials of probiotics for prevention and treatment of pediatric atopic dermatitis. J. Allergy Clin. Immunol. 121, 116–121 e11. 10.1016/j.jaci.2007.12.290 18206506

[B76] Licciardi P. V. Tang M. L. (2011). Vaccine adjuvant properties of probiotic bacteria. Discov. Med. 12, 525–533 22204769

[B77] Licciardi P. V. Wong S. S. Tang M. L. Karagiannis T. C. (2010). Epigenome targeting by probiotic metabolites. Gut Pathog. 2, 24 10.1186/1757-4749-2-24 21172038 PMC3016256

[B78] Lopez P. Gueimonde M. Margolles A. Suarez A. (2010). Distinct Bifidobacterium strains drive different immune responses in vitro. Int. J. Food Microbiol. 138, 157–165 10.1016/j.ijfoodmicro.2009.12.023 20071048

[B79] Macia L. Thorburn A. N. Binge L. C. Marino E. Rogers K. E. Maslowski K. M. Vieira A. T. Kranich J. Mackay C. R. (2012). Microbial influences on epithelial integrity and immune function as a basis for inflammatory diseases. Immunol. Rev. 245, 164–176 10.1111/j.1600-065X.2011.01080.x 22168419

[B80] Macpherson A. J. Hunziker L. McCoy K. Lamarre A. (2001). IgA responses in the intestinal mucosa against pathogenic and non-pathogenic microorganisms. Microbes Infect. 3, 1021–1035 10.1016/S1286-4579(01)01460-5 11580989

[B81] Majamaa H. Isolauri E. (1997). Probiotics: a novel approach in the management of food allergy. J. Allergy Clin. Immunol. 99, 179–185 10.1016/S0091-6749(97)70093-9 9042042

[B82] Maldonado Galdeano C. Novotny Nunez I. de Moreno de LeBlanc A. Carmuega E. Weill R. Perdigon G. (2011). Impact of a probiotic fermented milk in the gut ecosystem and in the systemic immunity using a non-severe protein-energy-malnutrition model in mice. BMC Gastroenterol. 11, 64 10.1186/1471-230X-11-64 21615956 PMC3125276

[B83] Marschan E. Kuitunen M. Kukkonen K. Poussa T. Sarnesto A. Haahtela T. Korpela R. Savilahti E. Vaarala O. (2008). Probiotics in infancy induce protective immune profiles that are characteristic for chronic low-grade inflammation. Clin. Exp. Allergy 38, 611–618 10.1111/j.1365-2222.2008.02942.x 18266878

[B84] Martin R. Nauta A. J. Ben Amor K. Knippels L. M. Knol J. Garssen J. (2010). Early life: gut microbiota and immune development in infancy. Benef. Microbes 1, 367–382 10.3920/BM2010.0027 21831776

[B85] Maslowski K. M. Vieira A. T. Ng A. Kranich J. Sierro F. Yu D. Schilter H. C. Rolph M. S. Mackay F. Artis D. Xavier R. J. Teixeira M. M. Mackay C. R. (2009). Regulation of inflammatory responses by gut microbiota and chemoattractant receptor GPR43. Nature 461, 1282–1286 10.1038/nature08530 19865172 PMC3256734

[B86] Miettinen M. Matikainen S. Vuopio-Varkila J. Pirhonen J. Varkila K. Kurimoto M. Julkunen I. (1998). Lactobacilli and streptococci induce interleukin-12 (IL-12), IL-18, and gamma interferon production in human peripheral blood mononuclear cells. Infect. Immun. 66, 6058–6062 9826398 10.1128/iai.66.12.6058-6062.1998 PMC108774

[B87] Mohan R. Koebnick C. Schildt J. Schmidt S. Mueller M. Possner M. Radke M. Blaut M. (2006). Effects of Bifidobacterium lactis Bb12 supplementation on intestinal microbiota of preterm infants: a double-blind, placebo-controlled, randomized study. J. Clin. Microbiol. 44, 4025–4031 10.1128/JCM.00767-06 16971641 PMC1698302

[B88] Molloy M. J. Bouladoux N. Belkaid Y. (2012). Intestinal microbiota: shaping local and systemic immune responses. Semin. Immunol. 24, 58–66 10.1016/j.smim.2011.11.008 22178452 PMC3292882

[B89] Mukai T. Asasaka T. Sato E. Mori K. Matsumoto M. Ohori H. (2002). Inhibition of binding of Helicobacter pylori to the glycolipid receptors by probiotic Lactobacillus reuteri. FEMS Immunol. Med. Microbiol. 32, 105–110 10.1111/j.1574-695X.2002.tb00541.x 11821231

[B90] Nagler-Anderson C. (2000). Tolerance and immunity in the intestinal immune system. Crit. Rev. Immunol. 20, 103–120 10.1615/CritRevImmunol.v20.i2.20 10872893

[B91] Niers L. Martin R. Rijkers G. Sengers F. Timmerman H. van Uden N. N. Smidt H. Kimpen J. Hoekstra M. (2009). The effects of selected probiotic strains on the development of eczema (the PandA study). Allergy 64, 1349–1358 10.1111/j.1398-9995.2009.02021.x 19392993

[B92] Niers L. E. Timmerman H. M. Rijkers G. T. van Bleek G. M. van Uden N. O. Knol E. F. Kapsenberg M. L. Kimpen J. L. Hoekstra M. O. (2005). Identification of strong interleukin-10 inducing lactic acid bacteria which down-regulate T helper type 2 cytokines. Clin. Exp. Allergy 35, 1481–1489 10.1111/j.1365-2222.2005.02375.x 16297146

[B93] Obata T. Goto Y. Kunisawa J. Sato S. Sakamoto M. Setoyama H. Matsuki T. Nonaka K. Shibata N. Gohda M. Kagiyama Y. Nochi T. Yuki Y. Fukuyama Y. Mukai A. Shinzaki S. Fujihashi K. Sasakawa C. Iijima H. Goto M. Umesaki Y. Benno Y. Kiyono H. (2010). Indigenous opportunistic bacteria inhabit mammalian gut-associated lymphoid tissues and share a mucosal antibody-mediated symbiosis. Proc. Natl. Acad. Sci. U.S.A. 107, 7419–7424 10.1073/pnas.1001061107 20360558 PMC2867693

[B94] Oelschlaeger T. A. (2010). Mechanisms of probiotic actions – a review. Int. J. Med. Microbiol. 300, 57–62 10.1016/j.ijmm.2009.08.005 19783474

[B95] Ohashi Y. Tokunaga M. Taketomo N. Ushida K. (2007). Stimulation of indigenous lactobacilli by fermented milk prepared with probiotic bacterium, Lactobacillus delbrueckii subsp. bulgaricus strain 2038, in the pigs. J. Nutr. Sci. Vitaminol. 53, 82–86 10.3177/jnsv.53.82 17484385

[B96] O’Keefe S. J. Ou J. Delany J. P. Curry S. Zoetendal E. Gaskins H. R. Gunn S. (2011). Effect of fiber supplementation on the microbiota in critically ill patients. World J. Gastrointest. Pathophysiol. 2, 138–145 10.4291/wjgp.v2.i6.138 22180847 PMC3240905

[B97] Osborn D. A. Sinn J. K. (2007). Probiotics in infants for prevention of allergic disease and food hypersensitivity. Cochrane Database Syst. Rev. 4, CD006475 17943912 10.1002/14651858.CD006475.pub2

[B98] Ouwehand A. C. Isolauri E. He F. Hashimoto H. Benno Y. Salminen S. (2001). Differences in Bifidobacterium flora composition in allergic and healthy infants. J. Allergy Clin. Immunol. 108, 144–145 10.1067/mai.2001.116003 11447399

[B99] Palomares O. Yaman G. Azkur A. K. Akkoc T. Akdis M. Akdis C. A. (2010). Role of Treg in immune regulation of allergic diseases. Eur. J. Immunol. 40, 1232–1240 10.1002/eji.200940045 20148422

[B100] Pelto L. Isolauri E. Lilius E. M. Nuutila J. Salminen S. (1998). Probiotic bacteria down-regulate the milk-induced inflammatory response in milk-hypersensitive subjects but have an immunostimulatory effect in healthy subjects. Clin. Exp. Allergy 28, 1474–1479 10.1046/j.1365-2222.1998.00449.x 10024217

[B101] Penders J. Thijs C. van den Brandt P. A. Kummeling I. Snijders B. Stelma F. Adams H. van Ree R. Stobberingh E. E. (2007). Gut microbiota composition and development of atopic manifestations in infancy: the KOALA Birth Cohort Study. Gut 56, 661–667 10.1136/gut.2006.100164 17047098 PMC1942165

[B102] Penders J. Thijs C. Vink C. Stelma F. F. Snijders B. Kummeling I. van den Brandt P. A. Stobberingh E. E. (2006). Factors influencing the composition of the intestinal microbiota in early infancy. Pediatrics 118, 511–521 10.1542/peds.2005-2824 16882802

[B103] Peng L. Li Z. R. Green R. S. Holzman I. R. Lin J. (2009). Butyrate enhances the intestinal barrier by facilitating tight junction assembly via activation of AMP-activated protein kinase in Caco-2 cell monolayers. J. Nutr. 139, 1619–1625 10.3945/jn.109.104638 19625695 PMC2728689

[B104] Pochard P. Gosset P. Grangette C. Andre C. Tonnel A. B. Pestel J. Mercenier A. (2002). Lactic acid bacteria inhibit TH2 cytokine production by mononuclear cells from allergic patients. J. Allergy Clin. Immunol. 110, 617–623 10.1067/mai.2002.128528 12373271

[B105] Prescott S. L. Bjorksten B. (2007). Probiotics for the prevention or treatment of allergic diseases. J. Allergy Clin. Immunol. 120, 255–262 10.1016/j.jaci.2007.03.045 17544096

[B106] Primeau M. N. Kagan R. Joseph L. Lim H. Dufresne C. Duffy C. Prhcal D. Clarke A. (2000). The psychological burden of peanut allergy as perceived by adults with peanut allergy and the parents of peanut-allergic children. Clin. Exp. Allergy 30, 1135–1143 10.1046/j.1365-2222.2000.00889.x 10931121

[B107] Rakoff-Nahoum S. Paglino J. Eslami-Varzaneh F. Edberg S. Medzhitov R. (2004). Recognition of commensal microflora by toll-like receptors is required for intestinal homeostasis. Cell 118, 229–241 10.1016/j.cell.2004.07.002 15260992

[B108] Rautava S. Arvilommi H. Isolauri E. (2006). Specific probiotics in enhancing maturation of IgA responses in formula-fed infants. Pediatr. Res. 60, 221–224 10.1203/01.pdr.0000228317.72933.db 16864708

[B109] Rautava S. Walker W. A. (2007). Commensal bacteria and epithelial cross talk in the developing intestine. Curr. Gastroenterol. Rep. 9, 385–392 10.1007/s11894-007-0047-7 17991339 PMC3208513

[B110] Reid G. (2005). The importance of guidelines in the development and application of probiotics. Curr. Pharm. Des. 11, 11–16 10.2174/1381612053382395 15638748

[B111] Resta-Lenert S. Barrett K. E. (2003). Live probiotics protect intestinal epithelial cells from the effects of infection with enteroinvasive Escherichia coli (EIEC). Gut 52, 988–997 10.1136/gut.52.7.988 12801956 PMC1773702

[B112] Riedler J. Braun-Fahrlander C. Eder W. Schreuer M. Waser M. Maisch S. Carr D. Schierl R. Nowak D. von Mutius E. (2001). Exposure to farming in early life and development of asthma and allergy: a cross-sectional survey. Lancet 358, 1129–1133 10.1016/S0140-6736(01)06252-3 11597666

[B113] Rimoldi M. Chieppa M. Larghi P. Vulcano M. Allavena P. Rescigno M. (2005). Monocyte-derived dendritic cells activated by bacteria or by bacteria-stimulated epithelial cells are functionally different. Blood 106, 2818–2826 10.1182/blood-2004-11-4321 16030185

[B114] Rosenfeldt V. Benfeldt E. Nielsen S. D. Michaelsen K. F. Jeppesen D. L. Valerius N. H. Paerregaard A. (2003). Effect of probiotic Lactobacillus strains in children with atopic dermatitis. J. Allergy Clin. Immunol. 111, 389–395 10.1067/mai.2003.389 12589361

[B115] Rupa P. Schmied J. Wilkie B. N. (2011). Prophylaxis of experimentally induced ovomucoid allergy in neonatal pigs using Lactococcus lactis. Vet. Immunol. Immunopathol. 140, 23–29 10.1016/j.vetimm.2010.11.004 21134696

[B116] Russell S. L. Gold M. J. Hartmann M. Willing B. P. Thorson L. Wlodarska M. Gill N. Blanchet M. R. Mohn W. W. McNagny K. M. Finlay B. B. (2012). Early life antibiotic-driven changes in microbiota enhance susceptibility to allergic asthma. EMBO Rep. 13, 440–447 10.1038/embor.2012.32 22422004 PMC3343350

[B117] Rutella S. Locatelli F. (2011). Intestinal dendritic cells in the pathogenesis of inflammatory bowel disease. World J. Gastroenterol. 17, 3761–3775 10.3748/wjg.v17.i33.3761 21987618 PMC3181437

[B118] Schiavi E. Barletta B. Butteroni C. Corinti S. Boirivant M. Di Felice G. (2011). Oral therapeutic administration of a probiotic mixture suppresses established Th2 responses and systemic anaphylaxis in a murine model of food allergy. Allergy 66, 499–508 10.1111/j.1398-9995.2010.02501.x 21058959

[B119] Setia A. Bhandari S. K. House J. D. Nyachoti C. M. Krause D. O. (2009). Development and in vitro evaluation of an Escherichia coli probiotic able to inhibit the growth of pathogenic Escherichia coli K88. J. Anim. Sci. 87, 2005–2012 10.2527/jas.2008-1400 19286822

[B120] Shaw M. H. Kamada N. Kim Y. G. Nunez G. (2012). Microbiota-induced IL-1beta, but not IL-6, is critical for the development of steady-state TH17 cells in the intestine. J. Exp. Med. 209, 251–258 10.1084/jem.20111703 22291094 PMC3280878

[B121] Shortt C. (1999). The probiotic century: historical and current perspectives. Trends Food Sci. Technol. 10, 411–417 10.1016/S0924-2244(99)00036-9

[B122] Shreffler W. G. Wanich N. Moloney M. Nowak-Wegrzyn A. Sampson H. A. (2009). Association of allergen-specific regulatory T cells with the onset of clinical tolerance to milk protein. J. Allergy Clin. Immunol. 123, 43–52 e7. 10.1016/j.jaci.2008.12.135 19130927

[B123] Sistek D. Kelly R. Wickens K. Stanley T. Fitzharris P. Crane J. (2006). Is the effect of probiotics on atopic dermatitis confined to food sensitized children? Clin. Exp. Allergy 36, 629–633 10.1111/j.1365-2222.2006.02485.x 16650048

[B124] Sjogren Y. M. Jenmalm M. C. Bottcher M. F. Bjorksten B. Sverremark-Ekstrom E. (2009). Altered early infant gut microbiota in children developing allergy up to 5 years of age. Clin. Exp. Allergy 39, 518–526 10.1111/j.1365-2222.2008.03156.x 19220322

[B125] Soh S. E. Aw M. Gerez I. Chong Y. S. Rauff M. Ng Y. P. Wong H. B. Pai N. Lee B. W. Shek L. P. (2009). Probiotic supplementation in the first 6 months of life in at risk Asian infants – effects on eczema and atopic sensitization at the age of 1 year. Clin. Exp. Allergy 39, 571–578 10.1111/j.1365-2222.2008.03133.x 19134020

[B126] Strachan D. P. (1989). Hay fever, hygiene, and household size. BMJ 299, 1259–1260 10.1136/bmj.299.6694.325 2513902 PMC1838109

[B127] Stratiki Z. Costalos C. Sevastiadou S. Kastanidou O. Skouroliakou M. Giakoumatou A. Petrohilou V. (2007). The effect of a bifidobacter supplemented bovine milk on intestinal permeability of preterm infants. Early Hum. Dev. 83, 575–579 10.1016/j.earlhumdev.2006.12.002 17229535

[B128] Su J. C. Kemp A. S. Varigos G. A. Nolan T. M. (1997). Atopic eczema: its impact on the family and financial cost. Arch. Dis. Child. 76, 159–162 10.1136/adc.76.2.159 9068310 PMC1717083

[B129] Sudo N. Sawamura S. Tanaka K. Aiba Y. Kubo C. Koga Y. (1997). The requirement of intestinal bacterial flora for the development of an IgE production system fully susceptible to oral tolerance induction. J. Immunol. 159, 1739–1745 9257835

[B130] Sui J. Leighton S. Busta F. Brady L. (2002). 16S ribosomal DNA analysis of the faecal lactobacilli composition of human subjects consuming a probiotic strain Lactobacillus acidophilus NCFM. J. Appl. Microbiol. 93, 907–912 10.1046/j.1365-2672.2002.01767.x 12392540

[B131] Talham G. L. Jiang H. Q. Bos N. A. Cebra J. J. (1999). Segmented filamentous bacteria are potent stimuli of a physiologically normal state of the murine gut mucosal immune system. Infect. Immun. 67, 1992–2000 10085047 10.1128/iai.67.4.1992-2000.1999 PMC96557

[B132] Tang M. L. (2009). Probiotics and prebiotics: immunological and clinical effects in allergic disease. Nestle Nutr. Workshop Ser. Pediatr. Program. 64, 219–235; discussion 235–238, 251–257. 10.1159/000235793 19710525

[B133] Tang M. L. Lahtinen S. J. Boyle R. J. (2010). Probiotics and prebiotics: clinical effects in allergic disease. Curr. Opin. Pediatr. 22, 626–634 20733491 10.1097/MOP.0b013e32833d9728

[B134] Tannock G. W. Munro K. Harmsen H. J. Welling G. W. Smart J. Gopal P. K. (2000). Analysis of the fecal microflora of human subjects consuming a probiotic product containing Lactobacillus rhamnosus DR20. Appl. Environ. Microbiol. 66, 2578–2588 10.1128/AEM.66.6.2578-2588.2000 10831441 PMC110584

[B135] Taylor A. L. Dunstan J. A. Prescott S. L. (2007). Probiotic supplementation for the first 6 months of life fails to reduce the risk of atopic dermatitis and increases the risk of allergen sensitization in high-risk children: a randomized controlled trial. J. Allergy Clin. Immunol. 119, 184–191 10.1016/j.jaci.2006.12.084 17208600

[B136] Tedelind S. Westberg F. Kjerrulf M. Vidal A. (2007). Anti-inflammatory properties of the short-chain fatty acids acetate and propionate: a study with relevance to inflammatory bowel disease. World J. Gastroenterol. 13, 2826–2832 17569118 10.3748/wjg.v13.i20.2826 PMC4395634

[B137] Todorov S. D. Botes M. Guigas C. Schillinger U. Wiid I. Wachsman M. B. Holzapfel W. H. Dicks L. M. (2008). Boza, a natural source of probiotic lactic acid bacteria. J. Appl. Microbiol. 104, 465–477 17922827 10.1111/j.1365-2672.2007.03558.x

[B138] Todorov S. D. Furtado D. N. Saad S. M. Gombossy de Melo Franco B. D. (2011). Bacteriocin production and resistance to drugs are advantageous features for Lactobacillus acidophilus La-14, a potential probiotic strain. New Microbiol. 34, 357–370 22143809

[B139] Tuomola E. Crittenden R. Playne M. Isolauri E. Salminen S. (2001). Quality assurance criteria for probiotic bacteria. Am. J. Clin. Nutr. 73(Suppl. 2), 393S–398S 11157347 10.1093/ajcn/73.2.393s

[B140] Umesaki Y. Setoyama H. (2000). Structure of the intestinal flora responsible for development of the gut immune system in a rodent model. Microbes Infect. 2, 1343–1351 10.1016/S1286-4579(00)01288-0 11018451

[B141] van de Pol M. A. Lutter R. Smids B. S. Weersink E. J. van der Zee J. S. (2011). Synbiotics reduce allergen-induced T-helper 2 response and improve peak expiratory flow in allergic asthmatics. Allergy 66, 39–47 10.1111/j.1398-9995.2010.02454.x 20716319

[B142] van der Aa L. B. Heymans H. S. van Aalderen W. M. Sillevis J. H. Smitt Knol J. Ben Amor K. Goossens D. A. Sprikkelman A. B. (2010). Effect of a new synbiotic mixture on atopic dermatitis in infants: a randomized-controlled trial. Clin. Exp. Allergy 40, 795–804 20184604 10.1111/j.1365-2222.2010.03465.x

[B143] van Nimwegen F. A. Penders J. Stobberingh E. E. Postma D. S. Koppelman G. H. Kerkhof M. Reijmerink N. E. Dompeling E. van den Brandt P. A. Ferreira I. Mommers M. Thijs C. (2011). Mode and place of delivery, gastrointestinal microbiota, and their influence on asthma and atopy. J. Allergy Clin. Immunol. 128, 948–955 e1–e3. 10.1016/j.jaci.2011.07.027 21872915

[B144] Vijay-Kumar M. Aitken J. D. Carvalho F. A. Cullender T. C. Mwangi S. Srinivasan S. Sitaraman S. V. Knight R. Ley R. E. Gewirtz A. T. (2010). Metabolic syndrome and altered gut microbiota in mice lacking Toll-like receptor 5. Science 328, 228–231 10.1126/science.1179721 20203013 PMC4714868

[B145] Viljanen M. Savilahti E. Haahtela T. Juntunen-Backman K. Korpela R. Poussa T. Tuure T. Kuitunen M. (2005a). Probiotics in the treatment of atopic eczema/dermatitis syndrome in infants: a double-blind placebo-controlled trial. Allergy 60, 494–500 10.1111/j.1398-9995.2004.00514.x 15727582

[B146] Viljanen M. Pohjavuori E. Haahtela T. Korpela R. Kuitunen M. Sarnesto A. Vaarala O. Savilahti E. (2005b). Induction of inflammation as a possible mechanism of probiotic effect in atopic eczema-dermatitis syndrome. J. Allergy Clin. Immunol. 115, 1254–1259 10.1016/j.jaci.2005.03.047 15940143

[B147] Wakabayashi H. Nariai C. Takemura F. Nakao W. Fujiwara D. (2008). Dietary supplementation with lactic acid bacteria attenuates the development of atopic-dermatitis-like skin lesions in NC/Nga mice in a strain-dependent manner. Int. Arch. Allergy Immunol. 145, 141–151 10.1159/000108139 17848807

[B148] Watanabe S. Narisawa Y. Arase S. Okamatsu H. Ikenaga T. Tajiri Y. Kumemura M. (2003). Differences in fecal microflora between patients with atopic dermatitis and healthy control subjects. J. Allergy Clin. Immunol. 111, 587–591 10.1067/mai.2003.1466 12642841

[B149] West C. E. Hammarstrom M. L. Hernell O. (2009). Probiotics during weaning reduce the incidence of eczema. Pediatr. Allergy Immunol. 20, 430–437 10.1111/j.1399-3038.2009.00745.x 19298231

[B150] Weston S. Halbert A. Richmond P. Prescott S. L. (2005). Effects of probiotics on atopic dermatitis: a randomised controlled trial. Arch. Dis. Child. 90, 892–897 10.1136/adc.2004.060673 15863468 PMC1720555

[B151] Wheeler J. G. Shema S. J. Bogle M. L. Shirrell M. A. Burks A. W. Pittler A. Helm R. M. (1997). Immune and clinical impact of Lactobacillus acidophilus on asthma. Ann. Allergy Asthma Immunol. 79, 229–233 10.1016/S1081-1206(10)63007-4 9305229

[B152] WHO. (2001). Health and Nutritional Properties of Probiotics in Food Including Powder Milk with Live Lactic Acid Bacteria. Available at: www.who.int/foodsafety/publications/fs_management/en/probiotics.pdf

[B153] Wickens K. Black P. N. Stanley T. V. Mitchell E. Fitzharris P. Tannock G. W. Purdie G. Crane J. (2008). A differential effect of 2 probiotics in the prevention of eczema and atopy: a double-blind, randomized, placebo-controlled trial. J. Allergy Clin. Immunol. 122, 788–794 10.1016/j.jaci.2008.07.011 18762327

[B154] Woo S. I. Kim J. Y. Lee Y. J. Kim N. S. Hahn Y. S. (2010). Effect of Lactobacillus sakei supplementation in children with atopic eczema-dermatitis syndrome. Ann. Allergy Asthma Immunol. 104, 343–348 10.1016/j.anai.2010.01.020 20408346

[B155] Wu K. G. Li T. H. Peng H. J. (2012). Lactobacillus salivarius plus fructo-oligosaccharide is superior to fructo-oligosaccharide alone for treating children with moderate to severe atopic dermatitis: a double-blind, randomized, clinical trial of efficacy and safety. Br. J. Dermatol. 166, 129–136 10.1111/j.1365-2133.2011.10666.x 21895621

[B156] Yang Y. J. Chuang C. C. Yang H. B. Lu C. C. Sheu B. S. (2012). Lactobacillus acidophilus ameliorates H. pylori-induced gastric inflammation by inactivating the Smad7 and NFkappaB pathways. BMC Microbiol. 12, 38 10.1186/1471-2180-12-38 22429929 PMC3340303

[B157] Yesilova Y. Calka O. Akdeniz N. Berktas M. (2012). Effect of probiotics on the treatment of children with atopic dermatitis. Ann. Dermatol. 24, 189–193 10.5021/ad.2012.24.2.189 22577270 PMC3346910

[B158] Yu J. Jang S. O. Kim B. J. Song Y. H. Kwon J. W. Kang M. J. Choi W. A. Jung H. D. Hong S. J. (2010). The effects of Lactobacillus rhamnosus on the prevention of asthma in a murine model. Allergy Asthma Immunol. Res. 2, 199–205 10.4168/aair.2010.2.3.199 20592920 PMC2892053

[B159] Zuercher A. W. Fritsche R. Corthesy B. Mercenier A. (2006). Food products and allergy development, prevention and treatment. Curr. Opin. Biotechnol. 17, 198–203 10.1016/j.copbio.2006.01.010 16481157

[B160] Zuercher A. W. Weiss M. Holvoet S. Moser M. Moussu H. van Overtvelt L. Horiot S. Moingeon P. Nutten S. Prioult G. Singh A. Mercenier A. (2012). Lactococcus lactis NCC 2287 alleviates food allergic manifestations in sensitized mice by reducing IL-13 expression specifically in the ileum. Clin. Dev. Immunol. 2012, 485750 10.1155/2012/485750 21961022 PMC3179883

[B161] Zyrek A. A. Cichon C. Helms S. Enders C. Sonnenborn U. Schmidt M. A. (2007). Molecular mechanisms underlying the probiotic effects of Escherichia coli Nissle 1917 involve ZO-2 and PKCzeta redistribution resulting in tight junction and epithelial barrier repair. Cell. Microbiol. 9, 804–816 10.1111/j.1462-5822.2006.00836.x 17087734

